# Black–White Differences in Housing Type and Sleep Duration as Well as Sleep Difficulties in the United States

**DOI:** 10.3390/ijerph15040564

**Published:** 2018-03-21

**Authors:** Dayna A. Johnson, Roland J. Thorpe, John A. McGrath, W. Braxton Jackson, Chandra L. Jackson

**Affiliations:** 1 Division of Sleep and Circadian Disorders, Brigham and Women’s Hospital, Harvard Medical School, Boston, MA 02131, USA; djohnson@research.bwh.harvard.edu; 2 Program for Research on Men’s Health, Hopkins Center for Health Disparities Solutions, Johns Hopkins Bloomberg School of Public Health, Baltimore, MD 21205, USA; rthorpe@jhu.edu; 3 Social & Scientific Systems, Inc., Research Triangle Park, NC 27709, USA; JMcgrath@s-3.com (J.A.M.); BJackson@s-3.com (W.B.J.); 4 Epidemiology Branch, National Institute of Environmental Health Sciences, National Institutes of Health, Department of Health and Human Services, 111 TW Alexander Drive, Research Triangle Park, NC 27709, USA

**Keywords:** sleep duration, sleep difficulties, housing, race, health disparities

## Abstract

Housing environments can directly and indirectly affect sleep, and blacks are more likely than whites to live in suboptimal housing conditions, which may independently contribute to sleep disparities. However, few large-scale epidemiological studies consider the potential influence of housing type on sleep health. Using data from the 2004–2015 National Health Interview Survey, we investigated overall and Black-White differences in the association between housing type (house/apartment versus mobile home/trailer) and sleep duration as well as sleep difficulties among 226,208 adults in the U.S. Poisson regression with robust variance was used to estimate sex-specific prevalence ratios (PR) for sleep categories, first comparing houses/apartments to mobile homes/trailers and then blacks to whites within housing types. All models were adjusted for age, educational attainment, income, occupational class, self-reported general health status, and region of residence. Compared to participants living in houses/apartments, the prevalence of short sleep was higher for men (PR = 1.05 (95% confidence interval (CI): 1.02–1.08)) and women (PR = 1.07 (95% CI: 1.04–1.09)) in mobile homes/trailers. Black men (PR = 1.26 (95% CI: 1.21–1.30)) and women (PR = 1.24 (95% CI: 1.20–1.27)) in a house/apartment were more likely to be short sleepers than their white counterparts. There was generally no significant difference in sleep characteristics (except long sleep) between black and white men in mobile homes/trailers after adjustments, and black men in houses/apartments as well as black women in both housing types were less likely to report sleep difficulties although being more likely to report short sleep. Overall, individuals in mobile homes/trailers, which may represent suboptimal housing, had worse sleep than those in houses/apartments; and racial differences in the quality of houses and apartments are likely to greatly vary in ways that still contribute to sleep disparities. Race–sex group differences in sleep duration among residents in a house/apartment and even a lack of racial difference among individuals living in mobile homes/trailers support the need for more research on residential environments and eventually multi-level interventions designed to reduce sleep disparities.

## 1. Introduction

The home environment—independent of neighborhoods—is an important, understudied determinant of health. Social and environmental exposures, such as safety and social cohesion, crowding, indoor air quality, inopportune light and noise, as well as other ambient conditions [1,2], which have been shown to disturb sleep [3,4,5,6], may vary by housing type, such as houses, apartments, and mobile homes. Suboptimal housing is also associated with poor health outcomes (e.g., type 2 diabetes; cardiovascular disease) that are often recognized as a consequence of or exacerbated by poor sleep [1,2,7,8,9]. Therefore, it is important to identify and understand the potential variation in sleep by housing type. Research on housing and sleep could help prioritize settings and approaches that may improve sleep and reduce the burden of subsequent poor health outcomes that often disproportionately affect racial minorities and other under-resourced populations.

Several epidemiologic studies have found that housing structure impacts sleep [10,11]. For instance, noisy home environments [12,13,14], household crowding [15,16], and suboptimal ambient temperature [17] were independently associated with insufficient sleep. These different housing characteristics likely vary by housing type and may provide important insights into how physical and social environments influence sleep duration and sleep difficulties. For instance, mobile home/trailer residents may have greater exposure to and less control over inopportune light, temperature, or noise compared to those residing in a house or apartment. Additionally, housing type may represent another marker of socioeconomic status, based on differences in housing structure integrity/quality, size, and aesthetics, which might be informed by the social and environmental conditions in which one dwells. Therefore, housing type may provide important insight regarding those most-at-risk for sleep problems. Also, investigating the role of housing type in relation to sleep patterns may provide a more comprehensive understanding of the impact of contextual factors on sleep.

Prior research regarding the home environment and its impact on health has been mainly conducted among non-Hispanic white adults or children [12,13,14,18], which limits generalizability to adults and racial/ethnic minorities who are disproportionately affected by poor sleep [19,20]. For example, blacks have been consistently shown to have a shorter sleep duration and poorer sleep quality than whites [19,21]. Additionally, blacks are particularly more likely than whites to reside in disadvantaged neighborhoods and have adverse housing conditions [22], which are known to contribute to insufficient sleep [23,24]. Therefore, it is plausible that sleep disparities are influenced by a distressed environment, such as suboptimal housing conditions (e.g., substandard structures, overcrowding) that varies by race. Based on prior evidence, which demonstrated that blacks generally have worse sleep than whites, a similar pattern is expected within housing types. Also, the racial disparity in sleep may be exacerbated in more optimal housing environments. Jackson and colleagues previously reported that a larger sleep disparity existed between blacks and whites of higher socioeconomic status than between those of lower socioeconomic status; thus, it is plausible that the racial sleep disparity may be greater in more optimal than suboptimal housing environments [20]. However, racial variations in sleep duration within different housing types in the United States (U.S.) are currently unknown.

Furthermore, racial disparities in sleep by housing type likely exist as a result of differential access to resources due to historical as well as contemporary discriminatory policies/practices [25]. To further understand disparities in sleep, it is important to investigate how sleep duration and sleep difficulties potentially vary by race within housing type. To address this important gap in the literature, we investigated racial differences in the association between housing type (specifically, house/apartment and mobile home/trailer, which is considered a particularly adverse environment) and self-reported sleep duration as well as sleep difficulties using a nationally representative sample of the U.S. We hypothesized that men and women living in a mobile home/trailer would have shorter (<7 h) and longer (≥9 h) sleep duration and report more sleep difficulties compared to those in a house/apartment; and black men and women would have shorter and longer sleep duration and report more sleep difficulties relative to white men and women in any housing type. The racial disparity was also hypothesized to be smaller among those in mobile homes/trailers. Our hypotheses included associations of both short and long sleep duration based on substantial evidence that has demonstrated a U-shaped relationship between sleep duration and adverse cardiovascular outcomes [26].

## 2. Methods

### 2.1. National Health Interview Survey

We analyzed data from the National Health Interview Survey (NHIS), which is a series of cross-sectional, nationally representative surveys that use a three-stage stratified cluster probability sampling design to conduct in-person interviews in the households of non-institutionalized U.S. civilians. A detailed description of NHIS procedures has been published elsewhere [27]. Briefly, a probability sample of households were interviewed by trained interviewers from the U.S. Census Bureau to obtain information about health and sociodemographic characteristics of the sampled household on a continual weekly basis. Data were collected using computer-assisted personal interviewing. A randomly selected adult and child (not included in this analysis) provided more specific health-related information. The final response rate for sample adults was 80.0% (range: 74.2–83.7%) for 2004–2015 surveys. The NHIS received written informed consent from each study participant.

### 2.2. Study Population

Study participants self-identified as Non-Hispanic white and Non-Hispanic black (hereafter, white and black) adults who were at least 18 years old. Participants were excluded if they were born outside the U.S. or had missing data on sleep measures or housing type. We excluded non-U.S. born participants because evidence suggests that sleep patterns among U.S. immigrants differ from those among individuals born in the U.S. [28]. Our final sample comprised 226,208 adults.

### 2.3. Measures

#### 2.3.1. Sleep Duration and Sleep Difficulties

Sampled adults reported how many hours they sleep, on average, in a 24-h period. By instruction, interviewers reported sleep hours in whole numbers while rounding values of 30 min or more up to the nearest hour and rounding values less than 30 min down to the nearest hour. Short sleep duration was defined as a usual sleep duration of less than 7 h; the recommended amount of sleep was defined as 7–8 h; and long sleep was defined as equal or more than 9 h. Seven-to-eight hours of sleep was used as the reference because it is associated with the lowest levels of morbidity and mortality [29]. These measures of sleep duration were available for 2004–2015. Additionally, several measures of sleep difficulties were assessed using questions from NHIS 2013–2015 on “trouble falling asleep”, “trouble staying asleep”, “waking up most days feeling rested”, and “took sleep medication one or more times” (all of these in the previous week).

#### 2.3.2. Housing Type

Housing type was assessed with twelve options as living quarters: “House, apartment, flat, condo”; “non-transient hotel, motel”; “permanent in transient hotel, motel”; “rooming house”; “Mobile home/trailer w/no permanent rooms added”; “Mobile home/trailer w/1+ permanent rooms added”; “not specified”; “Quarters not housing unit in room or board house”; “Unit not permanent-transient hotel, motel”; “Unoccupied site for mobile home/trailer/tent”; “Student quarters in college dormitory”; and “Group quarter unit not specified above”. The above options were then categorized into four types of housing: “house, apartment/condo”, “mobile home/trailer”, “dormitory”, and “hotel”. This analysis focused on the “house, apartment/condo” (*N* = 213,874) and “mobile home/trailer” (*N* = 12,334) housing types because the dormitory setting included one demographic of young adult college students and the sample size for hotel was too small for robust estimation.

#### 2.3.3. Race/Ethnicity

Participants were asked, “What race or races do you consider yourself to be?” They then selected ≥1 of the following categories: American Indian/Alaskan native, Asian, black/African American, white, or multiple races. National origin or ancestry refers to the national or cultural group from which the person is descended, and ethnicity is classified as Hispanic or Non-Hispanic. This study focuses on blacks and whites because the largest disparity is often observed between these groups and the underlying social as well as biological (not necessarily genetic) mechanisms leading to differences in sleep duration and subsequent health outcomes are likely to vary by race/ethnicity [25,30]. Whites were used as the comparison group for greater statistical stability because this group has the largest sample size.

#### 2.3.4. Demographic and Clinical Characteristics

*Socioeconomic status*. Socioeconomic status (SES) was based on education, income, and occupational class. Educational attainment was categorized as less than high school (no high school diploma), high school (high school or general equivalency diploma), some college (any education beyond high school), and college graduate and beyond. Annual household income was dichotomized as below $35,000 versus $35,000 or above. Adults who were working at a paying or nonpaying job during the week prior to the survey, those who had a job or business but were not at work during the prior week, and those who had ever worked were asked about their occupations, which were categorized on the basis of the Standard Occupational Classification System (http://www.bls.gov/soc/). We combined occupations into categories of professional/management, support services, or laborers based on type of work. Class of work/occupation (based on current, longest held, or most recently held job or work situation) was classified as either (1) an employee of a private company, business, or individual for wages, salary, or commission; (2) a federal, state, or local government employee; (3) self-employed in own business, professional practice, or farm; or (4) working without pay in a family-owned business or farm. Furthermore, marital status was categorized as married/living with a partner, divorced/separated/widowed, or never married.

*Region of residence.* Regions of the country were categorized as South, Midwest, Northeast, or West.

*Clinical characteristics*. Self-reported height and weight were used to calculate body mass index (BMI). Obesity was defined as a BMI of ≥30 kg/m^2^, overweight as 25.0–29.9 kg/m^2^, normal weight as 18.5–24.9 kg/m^2^, and underweight as less than 18.5 kg/m^2^ [31]. Self-reported general health status was categorized as “excellent/very good”, “good”, or “fair/poor”.

#### 2.3.5. Statistical Analysis

We pooled 12 survey years (2004–2015) of NHIS data merged by the Integrated Health Interview Series. For all analyses, we used a stratification variable and sampling weights that account for the unequal probabilities of selection resulting from the sample design, nonresponse, and oversampling of certain subgroups. Standard errors or variance estimates were calculated by using Taylor series linearization [32]. Stata, version 14, software (StataCorp. LP, College Station, TX, USA) was used for all analyses.

Categorical variables are presented using raw (unweighted) frequencies accompanied by the weighted percentages; additionally, all percentages were standardized to the age structure of the 2010 Census (with three age groups: 18–49, 50–64, and 65+). We used Rao Scott second-order corrected Pearson statistics that take survey weights into account to test for differences in pre-specified sociodemographic, clinical, and behavioral characteristics of interest between whites and blacks, as well as by categories of sleep outcomes.

We calculated prevalence ratios (PRs) comparing housing types as well as blacks and whites on sleep duration and sleep difficulties as a function of housing type; we used Poisson regression with a robust variance estimator and included sampling weights [33]. Pre-specified socioeconomic, demographic, and clinical characteristics were entered into the model as a group, and white participants were used as the reference for the black–white comparisons. We adjusted for age in three categories (18–49 years, 50–64 years, or ≥65 years) for socioeconomic and demographic factors (income, marital status, educational attainment, and region of residence) and for self-reported health status. Because depression was only available for 2013–2015, it was not included as a potential confounder in regression models. In a secondary data analyses, we additionally adjusted for obesity, type 2 diabetes, and hypertension.

Among men and women separately, we estimated sleep outcomes comparing blacks and whites within housing types.

## 3. Results

Sociodemographic, health behavior, clinical, and healthcare access/utilization characteristics of men and women are shown by housing type and race in Table 1. Of the 226,208 black and white participants, 55% were female, and 6% of whites and 4% of blacks lived in a mobile home/trailer. Blacks were generally younger than whites across housing type, and were more likely to live in poverty even among individuals in mobile homes/trailers.

### 3.1. Sociodemographic and Sleep Characteristics by Race and Sex for Houses/Apartments

Among men who lived in a house/apartment, black men were more likely than white men to have a less than high school education (17% versus 8%), an annual household income less than $35,000 (45% versus 24%), an occupational status of laborer (63% versus 44%), reside in the South (59% versus 33%), and have fair/poor self-reported general health status (21% versus 11%). Black men were also more likely than white men to be short (36% versus 27%) and long (11% versus 8%) sleepers as well as less likely to obtain the recommended amount of sleep (54% versus 65%).

Black women living in a house or apartment were more likely than their white female counterparts to have a less than high school education (16% versus 7%), an annual household income less than $35,000 (56% versus 30%), an occupational status of laborer (29% versus 16%), reside in the South (59% versus 34%), and a fair/poor general health status (23% versus 12%). Black women were also more likely than white women to be short (37% versus 27%) and long (11% versus 9%) sleepers as well as less likely to obtain the recommended amount of sleep (52% versus 64%).

### 3.2. Sociodemographic and Sleep Characteristics by Race and Sex Living in a Mobile Home/Trailer

Among participants who live in a mobile home/trailer, black men were more likely than their white counterparts to have a less than high school education (34% versus 24%), an annual household income <$35,000 (68% versus 57%), an occupational status of laborer (84% versus 75%), a residence in the South (95% versus 55%), and a fair/poor self-reported health status (35% versus 27%). White men living in mobile homes/trailers were more likely to be short sleepers (34% versus 29%) but less likely (12% versus 19%) to be long sleepers compared to black men. Black men were generally as likely to report the recommended amount of sleep (52% versus 54%) as white men.

Black women were more likely than white women to have a less than high school education (29% versus 23%), have an annual household income less than $35,000 (74% versus 62%), have an occupational status of laborer (54% versus 38%), a residence in the South (96% versus 57%), and health status reported as fair/poor (34% versus 28%) than white women. While black women were equally likely to report the recommended amount of sleep (53% versus 51%) as white women, white women living in mobile homes/trailers were more likely to be short sleepers (37% versus 31%) but less likely to be long sleepers (12% versus 16% for women) compared to their black counterparts.

### 3.3. Prevalence Ratios of Sleep by Housing Type in the Overall Population

Fully adjusted prevalence ratios for sleep duration and difficulties by housing type are shown in Table 2. The adjusted prevalence of short sleep was higher for men (PR = 1.05 (95% confidence interval (CI): 1.02–1.08)) and women (PR = 1.07 (95% CI: 1.04–1.09)) in mobile homes/trailers compared to those living in houses/apartments. Long sleep duration was more prevalent among men (PR = 1.09 (95% CI: 1.03–1.15)) but not women (PR = 1.03 (95% CI: 0.98–1.08)) in mobile homes/trailers compared to their counterparts in houses/apartments. In models additionally adjusted for obesity, type 2 diabetes, and hypertension, the effect estimates were similar, and thus the results did not appreciably change (Supplementary Materials Table S1). There were no differences in reported sleep difficulties between men and women in mobile homes/trailers compared to those in homes/apartments.

### 3.4. Prevalence Ratios of Sleep by Race and Housing Type

The adjusted prevalence ratios for racial differences in sleep duration and difficulties by housing type for men and women are displayed in Table 3. Compared to white men who live in a house/apartment, the adjusted prevalence was 26% (PR = 1.26 (95% CI: 1.21–1.30)) higher among black men who reported <7 h of sleep and 17% higher among those who reported >8 h of sleep (PR = 1.17 (95% CI: 1.09–1.27)). Also, black men who lived in a mobile home/trailer had a 36% (PR = 1.36 (95% CI: 1.11–1.67)) higher prevalence of sleeping >8 h relative to white men who lived in a mobile home/trailer. There were no differences observed between white and black men who live in a mobile home/trailer as it relates to reporting <7 h of sleep.

Black women who lived in a house/apartment had a 24% (PR = 1.24 (95% CI: 1.20–1.27)) higher prevalence of reporting sleep <7 h relative to white women who lived in a house/apartment. There was no difference in reporting >8 h of sleep between black and white women who live in a house/apartment. Similarly, the adjusted prevalence of reporting <7 h of sleep (PR = 0.89 (95% CI: 0.74–1.06)) or >8 h of sleep (PR = 1.19 (95% CI: 0.93–1.53)) was not different between black and white women who lived in a mobile home/trailer.

Regarding sleep difficulties, compared to white men who lived in a house/apartment, black men had a lower adjusted prevalence of having trouble falling asleep (PR = 0.84 (95% CI: 0.78–0.90)) and having trouble staying asleep (PR = 0.85 (95% CI: 0.80–0.91)), but had a higher adjusted prevalence of reporting waking up feeling rested on most days (PR = 1.05 (95% CI: 1.01–1.09)). Black men were also less likely to take a sleep medication at least once in the prior week (PR = 0.71 (95% CI: 0.61–0.82)) than white men. Among men who lived in a mobile home/trailer, black men had a lower adjusted prevalence of having trouble staying asleep (PR = 0.71 (95% CI: 0.51–0.99)) than white men, but there were no statistically significant differences in the adjusted prevalence of having trouble falling asleep, waking up feeling rested on most days, or reporting frequency of taking a sleep medication in the last week one or more times.

Compared to white women who resided in a house/apartment, their black counterparts reported a significantly lower adjusted prevalence of having trouble falling asleep (PR = 0.84 (95% CI: 0.80–0.89)) and having trouble staying asleep (PR = 0.82 (95% CI: 0.78–0.86)), but had a higher adjusted prevalence of reporting waking up feeling rested on most days (PR = 1.06 (95% CI: 1.02–1.10)). Black women were also less likely to take a sleep medication in the prior week (PR = 0.67 (95% CI: 0.61–0.73)) compared to white women in a house/apartment. A comparable pattern was observed for women who lived in a mobile home/trailer. For example, black women had a lower adjusted prevalence of having trouble falling asleep (PR = 0.77 (95% CI: 0.64–0.94)) and having trouble staying asleep (PR = 0.73 (95% CI: 0.56–0.95)), but had a 59% (PR = 1.59 (95% CI: 1.30–1.94)) higher prevalence of reporting waking up feeling rested most of the days compared to white women who also lived in a mobile home/trailer. Black women compared to white women in mobile homes/trailers were also less likely to take sleep medications in the prior week (PR = 0.33 (95% CI: 0.22–0.50)).

In secondary analyses, additional adjustment for obesity, type 2 diabetes, and hypertension resulted in similar effect estimates, which did not appreciably change the results (Supplementary Materials Table S2).

## 4. Discussion

In a nationally representative sample of black and white U.S. adults, we found important disparities in sleep duration and sleep difficulties by housing type overall as well as by race within housing type among both men and women. Our results support the idea that residing in a home or apartment is generally better for sleep duration than residing in mobile homes or trailers. For instance, individuals who lived in mobile homes/trailers were more likely to have shorter sleep duration and report fair/poor health in comparison to those who lived in a house or apartment. Before adjustments, short sleep duration was more prevalent among blacks compared to whites living in a house/apartment and more prevalent among whites compared to blacks living in mobile homes/trailers. After adjustments, there were striking racial disparities in sleep duration among those who lived a house or apartment that remained. Black men and women who resided in a house or apartment were more likely to report a habitual short sleep duration and were more likely to report their health as fair or poor compared to their white counterparts. However, no significant racial difference was observed for those in mobile homes/trailers with the exception of black men, who were significantly more likely than white men to report long sleep duration. Black men and women who lived in homes or apartments had a lower prevalence of sleep difficulties and were more likely to report waking feeling rested relative to their white counterparts. These black/white differences in sleep difficulties did not apply to men in trailers/mobile homes as there was no significant difference except among black men who were less likely to report trouble staying asleep. Black women in a mobile home/trailer had a lower prevalence of sleep difficulties and a higher prevalence of feeling rested most days compared to their white counterparts. Given the racial disparities in housing type, it is important to deeply understand these findings as a potential effective approach to improve sleep health and address health disparities.

Although the scientific evidence describing the impact of housing conditions on sleep is sparse, individuals who reside in mobile homes/trailers likely have greater exposure to adverse housing and neighborhood conditions that can negatively impact health compared to individuals who reside in houses/apartments. Mobile homes tend to be concentrated in more economically deprived neighborhoods, and prior studies have shown that deprived neighborhoods with crime, violence, or disorder are associated with shorter sleep duration [3,23]. More studies need to also assess the impact on sleep quality. While the mechanisms underlying the association are unclear, mobile homes/trailers may represent a particularly vulnerable/adverse environment where residents are more likely to experience stressors (e.g., housing and other financial-related insecurities; safety concerns) that impact sleep compared to those in more relatively stable environments, such as houses or apartments. These individuals may also be more likely to have overt or even preclinical health conditions that affect or are affected by suboptimal sleep.

Both black men and women reported shorter sleep duration relative to white men and women living in houses and apartments. The observed racial disparity in sleep duration may be attributable to various factors. For instance, black residents may experience more perceived stress, which is associated with short sleep duration [23]. In the current study sample, black compared to white participants were less likely to be homeowners, and thus may represent either a more transient or lower SES population, which is also independently associated with short sleep duration [34]. There was no racial difference in short sleep duration among those in mobile homes/trailers. Blacks who reside in homes or apartments tend to be of higher SES than those living in mobile homes, and a study by Jackson et al. found that blacks of higher SES appear more likely to be short sleepers than their lower SES counterparts [20]. Moreover, data suggest that highly educated blacks may be more vulnerable to the effects of stress on sleep duration than blacks with a lower level of educational attainment [34]. There are additional data suggesting that health behaviors (e.g., alcohol consumption) and chronic conditions (e.g., obesity, hypertension) are similar when blacks and whites live in similar environments [35,36,37,38]. Furthermore, disparities in wealth as well as access to positive material and intangible resources are likely to be much wider among blacks and whites who live in houses and even apartments than in mobile homes as the quality of the housing structures and surrounding amenities likely differ greatly by SES and race. Regarding individuals in houses/apartments, the percentage reporting low income was almost double for black versus white men, and black versus white women; this black–white gap in low-income was reduced in mobile homes/trailers, although poverty was worse. Thus, we expected to observe that the racial disparity in sleep would be lessened in mobile homes/trailers.

In both homes/apartments and mobile homes/trailers, black men were significantly more likely than white men to report long sleep duration. Prior studies using data from nationally representative samples have also demonstrated that black adults have a higher prevalence of both short and long sleep durations, which may indicate more variation in habitual sleep time [39]. This finding has significant implications regarding health outcomes. There are links between long sleep duration and increased mortality and incident cardiovascular disease [40]. Although the levels of clinical significance have not been established regarding sleep duration, the observed prevalence estimates of short and long sleep duration comparing black and white adults were similar to the effect sizes for well-known associations, such as short sleep duration and various cardiovascular outcomes (diabetes (odds ratio (OR) = 1.37, 95% CI 1.22–1.53), hypertension (1.17, 1.09–1.26), obesity (1.38, 1.25–1.53)) [41]. Future research should explore features of the housing environment that may promote suboptimal sleep durations among black man, particularly in efforts to reduce subsequent adverse health outcomes.

Regarding racial differences in reports of sleep difficulties, blacks have been shown to be less likely to self-report sleep complaints [42], but their sleep is consistently worse than whites based on objective sleep measures [43]. Future studies should investigate whether the observed finding that black men and women report less sleep difficulties is an accurate finding or due to misclassification. If due to misclassification, objective measures of sleep quality will be necessary in future studies. Further, disparities in sleep difficulties may be patterned differently among lower SES blacks and whites. Lower SES whites who reside in mobile homes may be more vulnerable to the effects of poor housing on sleep because whites are less likely to have experienced historical disadvantage related to concentrated poverty [44]. Studies have demonstrated that white men and women relative to their black counterparts tend to be less resilient to adversities due to the infrequent experiences of stressors, which have been shown to have a stronger impact on physical and mental health [44,45,46]. In the current study, more white men and women reported feeling depressed than their black counterparts in mobile homes, which supports the notion that white men and women may be more (or more likely to report being) mentally affected by their disadvantaged circumstance. Black individuals are a historically disadvantaged population across multiple generations, and research has shown that individuals can become desensitized and adopt behavioral techniques (e.g., alcohol and other substance abuse) in an attempt to buffer the negative mental health consequences with cumulative exposures to multiple stressors [47], which may help to explain this finding. Nonetheless, future studies are needed to better understand these relationships.

Our study has several limitations. This was a cross-sectional analysis, which precludes inferences related to causation because of difficulty in establishing temporality, avoiding reverse causation and potential endogeneity bias, and measuring the dynamic nature of neighborhoods as well as more immediate housing conditions (e.g., poor/substandard quality structures, overcrowding, unstable neighborhood). Furthermore, our sleep duration measure was based on self-report, which is prone to measurement error as individuals generally tend to overestimate their sleep duration [48]. Due to the NHIS data collection methods, we were also unable to differentiate between living in a house versus an apartment. These dwellings likely have differential social and environmental exposures that impact health. Additionally, there were fewer participants who resided in a mobile home/trailer compared to a house/apartment; however, the sample size was sufficient to provide robust estimates for our main research question. Furthermore, there are important potential confounders, such as psychosocial stress and urban density, that were unmeasured and several important confounders (e.g., income) were adjusted for in a crude manner, which could result in residual confounding. We were also unable to assess housing insecurity or level of mobility, which likely varies by housing type. We also could not assess endogeneity and did not include institutionalized (e.g., nursing homes) populations.

Despite the limitations, this study has important strengths. For instance, these data are based on a nationally representative large sample of black and white men and women, which allowed for robust estimation despite stratification. We also provided new data regarding racial differences in sleep duration among those who reside in apartments/houses, which helps to identify vulnerable populations that need tailored interventions focused on reducing sleep disparities. Furthermore, we included directly estimated prevalence ratios rather than the more difficult to interpret odds ratios, which simply approximate prevalence ratios. Lastly, the analysis included a large number of serial sleep measurements across survey years from 2004 to 2015.

## 5. Conclusions

In conclusion, these findings demonstrate that individuals in mobile/trailer homes, which could serve as a marker of suboptimal housing, may have poorer sleep than those in houses/apartments. Furthermore, there were also important within and between race–sex group differences in sleep duration and difficulties by housing type that need to be further investigated. The physical and social features of housing- and neighborhood-level factors that promote poor sleep need to be illuminated to eventually develop tailored intervention strategies at multiple levels that improve sleep in the population while addressing disparities.

## Figures and Tables

**Table 1:** Sociodemographic, Health Behavior, Clinical, and Healthcare Access/Utilization Characteristics among U.S. Adults by Housing Type Stratified by Gender, National Health Interview Survey, 2004–2015 (*N* = 226,208).

	Men (*N* = 102,108)								Women (*N* = 124,100)							
Characteristic ^a^	House/Apartment				Mobile Home				House/Apartment				Mobile Home			
	White	%	Black	%	White	%	Black	%	White	%	Black	%	White	%	Black	%
Sample size	80,857	94	15,410	96	5083	6	758	4	93,812	94	23,795	96	5554	6	939	4
Age, year, mean ± SE	49.7	6	47.7	14	50.5	24	49.2	58	51.5	6	47.4	12	51.3	25	46.3	54
Educational attainment																
<High school	6153	8	2479	17	1141	24	245	34	6900	7	3821	16	1226	23	221	29
High school graduate	22,678	29	5671	37	2336	46	351	44	26,135	29	7585	32	2336	44	401	43
Some college	24,568	29	4645	29	1279	24	138	20	30,726	33	8000	33	1586	27	268	24
≥College	27,230	34	2525	16	298	6	17	3	29,792	31	4253	18	386	6	44	4
Marital status																
Married	42,684	64	5120	46	2031	51	262	46	44,128	58	4662	29	2087	48	229	33
Divorced/separated/widowed	17,485	16	4443	24	1899	30	271	30	32,324	26	9083	38	2645	39	367	38
Never married	20,521	20	5795	30	1136	19	224	24	17,103	16	9928	33	800	13	338	28
Unemployed	26,542	34	6595	46	2365	48	372	53	41,459	44	10,987	49	3119	57	442	54
Annual Household income (<$35,000 per year)	22,101	24	2943	45	7324	57	510	68	31,831	30	13,997	56	3559	62	687	74
Living in poverty	5874	6	2748	17	880	18	211	30	8839	8	6572	26	1267	22	354	38
Occupation																
Professional/management	21,327	28	1650	11	479	10	26	4	15,011	17	2392	12	376	7	37	4
Support Services	21,580	28	3775	26	719	16	80	11	58,258	67	12,978	59	2898	55	374	42
Laborers	34,379	44	8664	63	3612	75	592	84	14,824	16	6061	29	1848	38	420	54
Home ownership	57,886	79	7345	58	3824	79	516	73	67,037	78	10,070	54	4185	79	658	73
Govt. Assistance	4398	5	2752	17	843	16	184	24	7890	7	7364	27	1337	23	359	34
Region of residence																
Northeast	14,215	19	1813	13	485	10	4	1	16,778	19	3210	14	515	9	4	1
Midwest	23,172	29	2927	19	948	17	16	2	26,847	29	4572	19	962	17	13	1
South	25,748	33	9016	59	2586	55	717	95	30,732	34	14,015	59	2919	57	903	96
West	17,722	19	1654	9	1064	18	21	2	19,455	18	1998	8	1158	17	19	2
Smoking status																
Never	38,960	48	8077	51	1500	29	333	44	54,250	58	15,876	66	2252	41	657	66
Former	24,456	32	3164	24	1457	30	145	21	22,232	24	3326	16	1173	22	100	15
Current	17,300	20	4123	25	2115	41	277	35	17,220	18	4553	18	2116	37	177	18
Alcohol consumption																
Never	8473	11	2978	20	587	12	150	20	17,776	19	8173	36	1428	27	413	46
Current	58,880	72	9251	58	3083	60	415	52	60,944	65	11,379	45	2704	48	323	29
Former	12,609	16	2928	22	1338	28	175	28	14,357	15	3981	19	1359	26	189	25
5+ drinks on at least 2 days ^b^	21,955	33	2619	26	1123	34	157	36	11,960	16	1468	11	548	18	46	12
Heavy drinking ^c^	17,135	24	1999	16	795	17	112	18	17,600	23	2128	13	500	12	59	10
Leisure-time physical activity																
Never/unable	22,934	29	5993	41	2468	50	409	56	29,485	31	11,264	49	2773	51	565	62
Low	27,643	35	4665	30	1199	23	181	24	31,009	34	6718	28	1339	24	201	19
High	29,926	36	4677	29	1388	27	164	21	33,050	35	5746	23	1424	25	171	20
Sad (past 30 days) (≥mostly)	1731	2	542	3	255	5	43	6	2816	3	1193	4	438	8	59	6
Felt depressed (often)	1436	7	274	7	150	15	16	8	2223	10	490	9	266	22	28	13
Felt depressed (a lot)	723	12	148	16	75	18	7	16	1375	15	345	19	164	27	21	24
Health outcomes																
Overweight prevalence ^d^	56,684	72	10,966	72	3524	71	539	72	49,592	53	17,271	74	3620	66	729	76
Obesity prevalence ^e^	20,902	27	4828	31	1566	32	251	33	22,634	24	9794	42	1963	36	460	48
Hypertension	26,146	35	6180	44	1999	42	343	49	29,149	31	10,337	50	2243	42	471	59
Diabetes	6947	10	2071	16	642	14	120	18	7161	8	3094	16	700	14	175	24
Cancer	8356	11	863	7	539	11	40	8	11,595	12	1252	6	812	14	49	7
Heart disease	11,026	15	1480	11	864	19	110	16	11,198	12	2447	12	882	16	94	12
Stroke	2342	3	695	5	271	6	42	6	3007	3	1070	5	295	5	62	11
Functional limitation	26,673	34	4940	35	2417	49	311	45	39,870	42	9942	46	3254	60	405	50
Any Injury (3 months)	2181	81	366	87	182	93	21	88	2923	87	608	91	236	96	21	89
Healthcare access and utilization																
No health insurance	9041	10	3082	18	1302	24	224	26	8041	8	3772	14	1162	21	227	22
Medicaid	2980	3	1836	11	539	10	126	18	6095	5	5397	19	987	16	233	22
Usual place of care	68,121	87	12,536	84	3928	79	607	84	86,085	93	21,518	92	4806	86	839	90
# ER visits in past year ≥2	4395	5	1554	10	502	10	105	15	6893	7	3357	13	808	15	159	17
Delayed healthcare due to cost	7951	8	1799	9	860	16	111	13	10,852	11	3240	12	1173	21	134	14
HIV testing (ever, yes)	24,829	29	8127	49	1603	31	304	40	31,233	31	13,065	49	2079	36	499	47
Health status																
Excellent/very good	51,854	63	7695	47	2192	41	288	37	59,262	63	11,161	43	2300	40	358	34
Good	19,975	25	4648	32	1594	32	218	28	23,501	25	7499	33	1799	33	302	32
Fair/poor	8990	11	3064	21	1296	27	251	35	11,007	12	5129	23	1453	28	278	34
Sleep duration																
<7 h	22,845	27	5678	36	1842	34	230	29	25,889	27	8759	37	2055	37	294	31
≥7–<9 h	51,640	65	8241	54	2697	54	392	52	59,285	64	12,594	52	2841	51	508	53
≥9 h	6372	8	1491	11	544	12	136	19	8638	9	2442	11	658	12	137	16
Trouble falling asleep	7518	30	1213	28	532	37	52	26	11,409	41	2375	37	730	50	93	37
Trouble staying asleep	9008	38	1340	32	612	42	53	28	12,798	47	2450	39	784	52	86	33
Most days woke up feeling rested	16,195	68	2882	69	856	60	145	66	16,761	62	3814	61	745	45	163	70
Times took sleep medication last week ≥1	3038	12	424	11	232	17	20	8	5325	19	909	14	361	26	31	10

NHIS = National Health Interview Survey; Data presented as mean ± standard error (SE) or n(); ^a^ Percentage may not sum to 100 due to missing values; ER = emergency room; ^b^ 5+ drinks on at least 2 days among men and women in 2013 and 4+ drinks; on at least 2 days among women in 2014 only; ^c^ heavy drinking = >2 drinks per day for men and >1 drink per day for women; ^d^ Overweight = ≥25 kg/m^2^; ^e^ Obesity = ≥25 kg/m^2^; HIV = Human Immunodeficiency Virus; Note. All estimates are weighted for the survey’s complex sampling design.

**Table 2:** Fully Adjusted Prevalence Ratios for Sleep Duration and Sleep Difficulties for Mobile Home/Trailer Residents Compared to People in Houses/Apartments, among U.S. Men and Women (Black and White): National Health Interview Survey, 2004–2015 (*N* = 226,208).

	House/Apartment	Mobile Home/Trailer	House/Apartment	Mobile Home/Trailer
	Men: Overall (*N* = 102,108)		Women: Overall (*N* = 124,100)	
Sleep duration				
<7 versus 7–8 h	1.0	1.05 (1.02–1.08)	1.0	1.07 (1.04–1.09)
≥9 versus 7–8 h	1.0	1.09 (1.03–1.15)	1.0	1.03 (0.98–1.08)
Trouble falling asleep (yes) *	1.0	1.01 (0.97–1.06)	1.0	1.02 (0.98–1.05)
Trouble staying asleep (yes) *	1.0	1.01 (0.97–1.06)	1.0	1.01 (0.98–1.05)
Days woke up feeling rested (most) *	1.0	0.98 (0.95–1.02)	1.0	0.92 (0.89–0.96)
Times took sleep medication last week ≥1) *	1.0	1.00 (0.93–1.08)	1.0	1.01 (0.95–1.07)

PR = Prevalence Ratio; CI = Confidence Interval; Models are adjusted for age, educational attainment, income, occupational class, health status, and region of residence. All estimates are weighted for the survey’s complex sampling design. Boldface indicates statistically significant results at the 0.05 level. * Data available from 2013–2015.

**Table 3:** Fully Adjusted Prevalence Ratios for Sleep Duration and Sleep Difficulties Compared to Housing Type among U.S. Black Men (referent White Men) and Black Women (referent White Women), National Health Interview Survey, 2004–2015 (*N* = 226,208).

	Men		Women	
	House/Apartment	Mobile Home/Trailer	House/Apartment	Mobile Home/Trailer
Sample size	Black: 15,410 White: 80,857	Black: 758 White: 5083	Black: 23,795 White: 93,812	Black: 939 White: 5554
Sleep duration				
<7 versus 7–8 h	1.26 (1.21–1.30)	0.90 (0.77–1.06)	1.24 (1.20–1.27)	0.89 (0.74–1.06)
≥9 versus 7–8 h	1.17 (1.09–1.27)	1.36 (1.11–1.67)	1.06 (1.00–1.13)	1.19 (0.93–1.53)
Trouble falling asleep (yes) *	0.84 (0.78–0.90)	0.83 (0.56–1.23)	0.84 (0.80–0.89)	0.77 (0.64–0.94)
Trouble staying asleep (yes) *	0.85 (0.80–0.91)	0.71 (0.51–0.99)	0.82 (0.78–0.86)	0.73 (0.56–0.95)
Days woke up feeling rested (most) *	1.05 (1.01–1.09)	1.10 (0.92–1.32)	1.06 (1.02–1.10)	1.59 (1.30–1.94)
Times took sleep medication last week ≥1) *	0.71 (0.61–0.82)	0.57 (0.30–1.09)	0.67 (0.61–0.73)	0.33 (0.22–0.50)

PR = Prevalence Ratio; CI = Confidence Interval; Adjusted for age, educational attainment, income, occupational class, health status, and region of residence. Note. All estimates are weighted for the survey’s complex sampling design. Boldface indicates statistically significant results at the 0.05 level. * data available from 2013–2015.

## Supplementary Materials

The following are available online at http://www.mdpi.com/1660-4601/15/4/564/s1, Table S1: Fully Adjusted Prevalence Ratios for Sleep Duration and Quality Measures for Mobile Home/Trailer Dwellers Compared to People in Houses/Apartments, among U.S. Men and Women (Black and White): National Health Interview Survey, 2004–2015 (*N* = 226,208), Table S2: Fully Adjusted Prevalence Ratios for Sleep Duration and Sleep Quality Indicators in Relation to Housing Type among U.S. Black Men (referent White Men) and Black Women (referent White Women), National Health Interview Survey, 2004–2015 (*N* = 226,208).

## References

[1] Shaw M. (2004). Housing and public health. Annu. Rev. Public Health.

[2] Krieger J., Higgins D.L. (2002). Housing and health: Time again for public health action. Am. J. Public Health.

[3] Johnson D.A., Simonelli G., Moore K., Billings M., Mujahid M.S., Rueschman M., Kawachi I., Redline S., Diez Roux A.V., Patel S.R. (2017). The Neighborhood Social Environment and Objective Measures of Sleep in the Multi-Ethnic Study of Atherosclerosis. Sleep.

[4] Johnson D.A., Brown D.L., Morgenstern L.B., Meurer W.J., Lisabeth L.D. (2015). The association of neighborhood characteristics with sleep duration and daytime sleepiness. Sleep Health.

[5] Hume K.I., Brink M., Basner M. (2012). Effects of environmental noise on sleep. Noise Health.

[6] Zanobetti A., Redline S., Schwartz J., Rosen D., Patel S., O’Connor G.T., Lebowitz M., Coull B.A., Gold D.R. (2010). Associations of PM_10_ with sleep and sleep-disordered breathing in adults from seven U.S. urban areas. Am. J. Respir. Crit. Care Med..

[7] Schootman M., Andresen E.M., Wolinsky F.D., Malmstrom T.K., Miller J.P., Yan Y., Miller D.K. (2007). The effect of adverse housing and neighborhood conditions on the development of diabetes mellitus among middle-aged African Americans. Am. J. Epidemiol..

[8] Morello-Frosch R., Zuk M., Jerrett M., Shamasunder B., Kyle A.D. (2011). Understanding the cumulative impacts of inequalities in environmental health: Implications for policy. Health Aff..

[9] Mullington J.M., Haack M., Toth M., Serrador J.M., Meier-Ewert H.K. (2009). Cardiovascular, inflammatory, and metabolic consequences of sleep deprivation. Prog. Cardiovasc. Dis..

[10] Chambers E.C., Pichardo M.S., Rosenbaum E. (2016). Sleep and the Housing and Neighborhood Environment of Urban Latino Adults Living in Low-Income Housing: The AHOME Study. Behav. Sleep Med..

[11] Simonelli G., Leanza Y., Boilard A., Hyland M., Augustinavicius J.L., Cardinali D.P., Vallières A., Pérez-Chada D., Vigo D.E. (2013). Sleep and quality of life in urban poverty: The effect of a slum housing upgrading program. Sleep.

[12] Solari C.D., Mare R.D. (2012). Housing crowding effects on children’s wellbeing. Soc. Sci. Res..

[13] Griefahn B., Marks A., Robens S. (2006). Noise emitted from road, rail and air traffic and their effects on sleep. J. Sound Vib..

[14] Jakovljevic B., Belojevic G., Paunovic K., Stojanov V. (2006). Road traffic noise and sleep disturbances in an urban population: Cross-sectional study. Croat. Med. J..

[15] Johnson D.A., Drake C., Joseph C.L., Krajenta R., Hudgel D.W., Cassidy-Bushrow A.E. (2015). Influence of neighbourhood-level crowding on sleep-disordered breathing severity: Mediation by body size. J. Sleep Res..

[16] Liu X., Liu L., Wang R. (2003). Bed sharing, sleep habits, and sleep problems among Chinese school-aged children. Sleep.

[17] Sandberg J.C., Talton J.W., Quandt S.A., Chen H., Weir M., Doumani W.R., Chatterjee A.B., Arcury T.A. (2014). Association between housing quality and individual health characteristics on sleep quality among Latino farmworkers. J. Immigr. Minor. Health.

[18] Chung S., Wilson K.E., Miller A.L., Johnson D.A., Lumeng J.C., Chervin R.D. (2014). Home Sleeping Conditions and Sleep Quality in Low-Income Preschool Children. Sleep Med. Res..

[19] Chen X., Wang R., Zee P., Lutsey P.L., Javaheri S., Alcántara C., Jackson C.L., Williams M.A., Redline S. (2015). Racial/Ethnic Differences in Sleep Disturbances: The Multi-Ethnic Study of Atherosclerosis (MESA). Sleep.

[20] Jackson C.L., Redline S., Kawachi I., Williams M.A., Hu F.B. (2013). Racial disparities in short sleep duration by occupation and industry. Am. J. Epidemiol..

[21] Hale L., Do D.P. (2007). Racial differences in self-reports of sleep duration in a population-based study. Sleep.

[22] Bureau U.S.C. (2013). American Housing Survey for the United States: 2011.

[23] Johnson D.A., Lisabeth L., Hickson D., Johnson-Lawrence V., Samdarshi T., Taylor H., Diez Roux A.V. (2016). The Social Patterning of Sleep in African Americans: Associations of Socioeconomic Position and Neighborhood Characteristics with Sleep in the Jackson Heart Study. Sleep.

[24] Desantis A.S., Diez Roux A.V., Moore K., Baron K.G., Mujahid M.S., Nieto F.J. (2013). Associations of neighborhood characteristics with sleep timing and quality: The Multi-Ethnic Study of Atherosclerosis. Sleep.

[25] Jackson C.L., Redline S., Emmons K.M. (2015). Sleep as a potential fundamental contributor to disparities in cardiovascular health. Annu. Rev. Public Health.

[26] Cappuccio F.P., Cooper D., D’Elia L., Strazzullo P., Miller M.A. (2011). Sleep duration predicts cardiovascular outcomes: A systematic review and meta-analysis of prospective studies. Eur. Heart J..

[27] National Center for Health Statistics CfDCaPNHISH, MD. http://www.cdc.gov/nchs/nhis.htm.

[28] Jackson C.L., Hu F.B., Redline S., Williams D.R., Mattei J., Kawachi I. (2014). Racial/ethnic disparities in short sleep duration by occupation: The contribution of immigrant status. Soc. Sci. Med..

[29] Watson N.F., Badr M.S., Belenky G., Bliwise D.L., Buxton O.M., Buysse D., Dinges D.F., Gangwisch J., Grandner M.A., Consensus Conference Panel (2015). Recommended Amount of Sleep for a Healthy Adult: A Joint Consensus Statement of the American Academy of Sleep Medicine and Sleep Research Society. J. Clin. Sleep Med..

[30] Jackson C.L. (2017). Determinants of racial/ethnic disparities in disordered sleep and obesity. Sleep Health.

[31] World Health Organization Expert Committee on Physical Status (1995). Use and Interpretation of Anthropometry. Physical Status: The Use and Interpretation of Anthropometry: Report of a WHO Expert Committee.

[32] Wolters K.M. (1990). Introduction to Variance Estimation.

[33] Barros A.J., Hirakata V.N. (2003). Alternatives for logistic regression in cross-sectional studies: An empirical comparison of models that directly estimate the prevalence ratio. BMC Med. Res. Methodol..

[34] Johnson D.A., Lisabeth L., Lewis T.T., Sims M., Hickson D.A., Samdarshi T., Taylor H., Diez Roux A.V. (2016). The Contribution of Psychosocial Stressors to Sleep among African Americans in the Jackson Heart Study. Sleep.

[35] Thorpe R.J., Kennedy-Hendricks A., Griffith D.M., Bruce M.A., Coa K., Bell C.N., Young J., Bowie J.V., LaVeist T.A. (2015). Race, Social and Environmental Conditions, and Health Behaviors in Men. Fam. Community Health.

[36] Fesahazion R.G., Thorpe R.J., Bell C.N., LaVeist T.A. (2012). Disparities in alcohol use: Does race matter as much as place?. Prev. Med..

[37] Bleich S.N., Thorpe R.J., Sharif-Harris H., Fesahazion R., Laveist T.A. (2010). Social context explains race disparities in obesity among women. J. Epidemiol. Community Health.

[38] LaVeist T., Pollack K., Thorpe R., Fesahazion R., Gaskin D. (2011). Place, not race: Disparities dissipate in southwest Baltimore when blacks and whites live under similar conditions. Health Aff..

[39] Nunes J., Jean-Louis G., Zizi F., Casimir G.J., von Gizycki H., Brown C.D., McFarlane S.I. (2008). Sleep duration among black and white Americans: Results of the National Health Interview Survey. J. Natl. Med. Assoc..

[40] Jike M., Itani O., Watanabe N., Buysse D.J., Kaneita Y. (2017). Long sleep duration and health outcomes: A systematic review, meta-analysis and meta-regression. Sleep Med. Rev..

[41] Itani O., Jike M., Watanabe N., Kaneita Y. (2017). Short sleep duration and health outcomes: A systematic review, meta-analysis, and meta-regression. Sleep Med..

[42] Grandner M.A., Patel N.P., Gehrman P.R., Xie D., Sha D., Weaver T., Gooneratne N. (2010). Who gets the best sleep? Ethnic and socioeconomic factors related to sleep complaints. Sleep Med..

[43] Lauderdale D.S., Knutson K.L., Yan L.L., Rathouz P.J., Hulley S.B., Sidney S., Liu K. (2006). Objectively measured sleep characteristics among early-middle-aged adults: The CARDIA study. Am. J. Epidemiol..

[44] Assari S., Lankarani M.M. (2016). Association between Stressful Life Events and Depression; Intersection of Race and Gender. J. Racial Ethn. Health Dispar..

[45] Assari S., Lankarani M.M. (2016). Depressive Symptoms Are Associated with More Hopelessness among White than Black Older Adults. Front. Public Health.

[46] Assari S. (2017). Race, sense of control over life, and short-term risk of mortality among older adults in the United States. Arch. Med. Sci..

[47] Jackson J.S., Knight K.M., Rafferty J.A. (2010). Race and unhealthy behaviors: Chronic stress, the HPA axis, and physical and mental health disparities over the life course. Am. J. Public Health.

[48] Lauderdale D.S., Knutson K.L., Yan L.L., Liu K., Rathouz P.J. (2008). Self-reported and measured sleep duration: How similar are they?. Epidemiology.

