# Supplementary material for: Black–White Differences in Housing Type and Sleep Duration as Well as Sleep Difficulties in the United States
Source: Int J Environ Res Public Health. 2018 Mar 21;15(4):564. doi: 10.3390/ijerph15040564 (PMC5923606; doi:10.3390/ijerph15040564)
Supplement: Supplementary file 1 [file ijerph-15-00564-s001.pdf]

International Journal of
Environmental Research and Public Health

MDPI

Supplementary Materials: Black-White Differences in Housing Type and Sleep Duration as well as Sleep Difficulties in the United States

Dayna A. Johnson [1], Roland J. Thorpe Jr. [2], John A. McGrath [3], W. Braxton Jackson II [3], and Chandra L. Jackson [4,*]

Table S1. Fully-Adjusted Prevalence Ratios for Sleep Duration and Quality Measures for Mobile Home/Trailer Dwellers Compared to People in Houses/Apartments, among U.S. Men and Women (Black and White): National Health Interview Survey, 2004-2015 (N = 226,208)

	**House/ Apartment**	**Mobile Home/Trailer**	**House/ Apartment**	**Mobile Home/Trailer**
	**Men – overall (n=102,108)**		**Women – overall (n=124,100)**	
Sleep duration				
<7 vs. 7-8 hours	1.0	**1.04 (1.02-1.07)**	1.0	**1.06 (1.04-1.09)**
>8 vs. 7-8 hours	1.0	**1.09 (1.03-1.15)**	1.0	1.02 (0.97-1.07)
Trouble falling asleep (yes)*	1.0	1.01 (0.97-1.06)	1.0	1.01 (0.98-1.05)
Trouble staying asleep (yes) *	1.0	1.01 (0.97-1.06)	1.0	1.01 (0.97-1.04)
Days woke up feeling rested (most)*	1.0	0.99 (0.96-1.02)	1.0	0.93 (0.89-0.96)
Times took sleep medication last week ≥1)*	1.0	1.00 (0.92-1.08)	1.0	1.01 (0.95-1.07)

PR=Prevalence Ratio; CI=Confidence Interval; Models are adjusted for age, educational attainment, income, occupational class, health status, obesity, type 2 diabetes, hypertension, and region of residence. All estimates are weighted for the survey's complex sampling design. Boldface indicates statistically significant results at the 0.05 level. *data available from 2013-2015.

Table S2. Fully-Adjusted Prevalence Ratios for Sleep Duration and Sleep Quality Indicators in Relation to Housing Type among U.S. Black Men (referent White Men) and Black Women (referent White Women), National Health Interview Survey, 2004-2015 (N = 226,208)

	**Men**		**Women**	
	House/ Apartment	Mobile Home/Trailer	House/ Apartment	Mobile Home/Trailer
Sample size	Black: 15,410 White: 80,857	Black: 758; White: 5,083	Black: 23,795; White: 93,812	Black: 939; White: 5,554
Sleep duration				
<7 v. 7-8 hours	1.25 (1.21-1.29)	0.90 (0.77-1.06)	1.21 (1.18-1.25)	0.87 (0.73-1.04)
>8 v. 7-8 hours	1.16 (1.08-1.26)	1.37 (1.12-1.69)	1.05 (0.99-1.11)	1.20 (0.93-1.56)
Trouble falling asleep (yes)*	0.83 (0.77-0.90)	0.83 (0.56-1.23)	0.83 (0.79-0.88)	0.73 (0.61-0.88)
Trouble staying asleep (yes) *	0.84 (0.79-0.90)	0.70 (0.50-0.98)	0.81 (0.77-0.85)	0.69 (0.53-0.91)
Days woke up feeling rested (most)*	1.06 (1.02-1.10)	1.12 (0.94-1.33)	1.07 (1.03-1.12)	1.62 (1.32-1.99)
Times took sleep medication last week ≥1)*	0.70 (0.60-0.82)	0.55 (0.27-1.11)	0.66 (0.60-0.72)	0.32 (0.21-0.49)

PR=Prevalence Ratio; CI=Confidence Interval; Adjusted for age, educational attainment, income, occupational class, health status, obesity, type 2 diabetes, hypertension, and region of residence. Note. All estimates are weighted for the survey's complex sampling design. Boldface indicates statistically significant results at the 0.05 level. *data available from 2013-2015. **NE: not estimable.

© 2017 by the authors. Submitted for possible open access publication under the terms and conditions of the Creative Commons Attribution (CC BY) license (http://creativecommons.org/licenses/by/4.0/).
